# Goodness of fit of the items used in the 2nd cycle of evaluation and accreditation of medical schools by the Korea Institute of Medical Education and Evaluation based on the Rasch model

**DOI:** 10.3352/jeehp.2019.16.28

**Published:** 2019-09-30

**Authors:** Man Sup Lim, Sun Huh

**Affiliations:** 1 Department of Medical Education, College of Medicine, Hallym University, Chuncheon, Korea; 2 Department of Parasitology and Institute of Medical Education, College of Medicine, Hallym University, Chuncheon, Korea; The Catholic University of Korea, Korea

**Keywords:** Medical education, Accreditation, Academies and institutes, Republic of Korea

## Abstract

**Purpose:**

Since 2004, the Korea Institute of Medical Education and Evaluation has been responsible for the evaluation and accreditation of medical schools in Korea. The 2nd cycle of evaluations was conducted from 2007 to 2011. The present study aimed at testing the goodness of fit of the items used in the 2nd cycle of evaluation and accreditation based on the Rasch model.

**Methods:**

Dichotomous data on 40 medical schools were analyzed using Winsteps, a tool based on the Rasch model that includes goodness-of-fit testing.

**Results:**

Two of the 109 items had an outfit mean square exceeding 2.0. The other 107 items showed a goodness of fit in the acceptable range for the outfit mean square. All items were in the acceptable range in terms of the infit mean square. Furthermore, 1 school had an outfit mean square exceeding 2.0, while all schools were in the acceptable range for the infit mean square. An outfit mean square value over 2.0 means that an item is a outlier. Therefore, 2 items showed an extreme response relative to the overall response. Meanwhile, the finding of an outfit mean square over 2.0 for 1 school means that it showed extraordinary responses to specific items, despite its excellent overall competency.

**Conclusion:**

The goodness of fit of the items used for evaluation and accreditation by the Korea Institute of Medical Education and Evaluation should be checked so that they can be revised appropriately. Furthermore, the outlier school should be investigated to determine why it showed such an inappropriate goodness of fit.

## Introduction

The Korea Institute of Medical Education and Evaluation (KIMEE) was recognized by the Ministry of Education of the Korean government as a medical education accreditation agency in 2014 and by the World Federation of Medical Education as the medical education accreditation agency in Korea in 2016. Before that, the Accreditation Board for Medical Education (ABMEK), which was established in 1998, conducted the 1st cycle of evaluations from 2000 to 2005 for 40 medical schools in Korea. Only 1 medical school did not participate in the 1st cycle evaluation. Then, KIMEE, which succeeded ABMEK, was founded with the approval of the Ministry of Health and Welfare in 2004. The 2nd cycle of evaluations was conducted from 2007 to 2011 for 40 medical schools. One medical school also did not participate in the 2nd cycle evaluation [1]. Subsequently, post-2nd cycle evaluations were done from 2012 to 2018 for 41 medical schools. The evaluation items used by an accreditation agency are critical for the validity and reliability of the evaluations. It is difficult to find psychometric studies on the evaluation and accreditation items used for medical schools in the literature, which may be due to the difficulties that researchers have accessing such data. Fortunately, data on the results of the 2nd cycle of evaluations conducted from 2007 to 2011 for 40 medical schools in Korea were obtained through the generosity of KIMEE. Therefore, it aimed to provide the psychometric characteristics of these items based on classical test theory and item response theory. Specifically followings were presented: first, response to 109 items by 40 medical schools according to classical test theory; second, correlation between the score obtained using classical test theory and measure (difficulty parameter) obtained using the Rasch model for 109 items.; third; Correlation between the scores obtained using classical test theory and measure (difficulty parameter) obtained using the Rasch model for 40 medical schools in Korea; and fourth, the goodness of fit tested based on the Rasch model, one of a well-known model within the framework of item response theory model [2]. Although the data are historical, this analysis will be able to provide evidence for the usefulness of the evaluation items used in the 2nd cycle of evaluations of medical schools in Korea by KIMEE.

## Methods

### Ethics statement

This study was approved by the Institutional Review Board of Hallym University with exemption from review and informed consent because the research data were not from human subjects (HIRB-2015-047).

### Study design

This was a descriptive study based on a psychometric analysis of the data collected during the 2nd cycle of the accreditation process of medical schools in Korea by KIMEE.

### Setting

From 2007 to 2011, reports from 40 medical schools in Korea were evaluated and the final decision regarding whether they fulfilled each of the 109 items was made after visits to 40 schools by medical educators trained in the accreditation process. From 2007 to 2011, there were 41 medical schools in Korea. Only 1 medical school did not participate in evaluation process by KIMEE; furthermore, this medical school was closed in 2018 by order of the Korean Government. Therefore, there were above 40 medical schools in September 2019 in Korea

### Data sources/measurement

Raw data on the fulfillment of the 109 items evaluated for 40 medical schools were received from KIMEE. Content validity of 109 items can be guaranteed because those items were developed by the specialists on medical education who work for KIMEE with a number of public hearings before implementation of evaluation process.

The 109 item data were classified into 41 required items, 34 recommended items, and 34 excellence (bonus) items. Raw data were converted to dichotomous data by treating marks with partial fulfillment or ‘not applicable’ as negative, such that only completely positive marks were treated as positive (Dataset 1). The content of each items is listed in Dataset 2. Unidimensionality was tested with 2 programs in DIMPACK [3]: DIMTEST and DETECT. Measurements of the data in both classical test theory and item response theory were made using Winsteps ver. 4.4.0 (Winsteps, Beaverton, OR, USA), which is a tool based on the Rasch model. The dichotomous data consisted of 109 items and 40 examinees’ responses. The control file of Winsteps, including its annotation, was as follows:

&INST; start statement
TITLE=“kimee3.con-Item analysis of KIMEE’s 2nd cycle evaluation of 40 medical schools in Korea from 2007 to 2011 by Sun Huh, Hallym University April 2, 2019” ; the title was an arbitrary description, File name “kimee3.con” is also arbitrarily created one.
PERSON=Med_schools ; the examinees were medical schools
ITEM=item ; each item is the title of ITEM
NI=109; number of items
ITEM1=1; column of responses to the first item in the data record
INUMB=Y ; the number of items was 109
XWIDE=1; number of columns per item response
CODES=10; valid codes in data file are 0 or 1 only
DATA=kimee3.dat ; name of dichotomous data file (Dataset 1) created by authors
IFILE=kimee3.IF ; name of item data file created by authors
PFILE=kimee3.PF ; name of person data file created by authors
CSV=Y ; a CSV file format of IFILE and PFILE is provided for use in computer spreadsheet programs
TABLES=111111111111111111111111111111 ; Tables from 1 to 30 are presented in the output file (Dataset 3)
&END ; closing statement

### Bias

There was no noteworthy source of bias during the collection of data or analysis process.

### Statistical methods

Descriptive and correlational analyses were carried out using DBSTAT ver. 5.0 (DBSTAT Co., Chuncheon, Korea).

## Results

### Unidimensionality test

The P-value obtained using DIMTEST was 0.1063 (Dataset 4), meaning that the hypothesis that the assessment subtest and partitioning subtest items were in the same dimension could not be rejected; therefore, all items were unidimensional. Furthermore, confirmatory analysis by DETECT showed a DETECT value of 0.0851. Because the DETECT value was less than 0.2, the data could be estimated as unidimensional (Dataset 5).

### Response to 109 items by 40 medical schools according to classical test theory

It was presented at the SCORE column in Dataset 6. Average score of 41 required items was 37.2. That of 34 recommended items was 34.9. That of 34 excellence (bonus) items was 16.3.

Out of required items, least fulfilled 2 items were as follows: Item 7: A person in charge of overseeing education and research must be appointed, and each hospital located separately must have an administrative structure that supports education and research. Regular meeting to standardize clinical training among different affiliated hospitals are held and actual actions have been taken according to meeting results (31 out of 40 medical schools fulfilled it). Item 10: The college must have a standing organization for continuous quality control and improvement of the college and conduct regular review on the college's organization and function (30 out of 40 medical schools fulfilled it).

Out of recommended items, least fulfilled 2 items were as follows: Item 75: The background of students are diverse enough to suit a specialized graduate school system and the internal/external scholarships or special support payments are at least 30% of the total paid tuition. There is a separate space exclusively for graduate student research (23 out of 40 medical schools fulfilled it). Item 64: The college must have at least 5 full-time faculties in medical humanities and social sciences (Half of medical schools fulfilled it).

Out of excellence items, least fulfilled items were as follows: Item 97: The domestic research result for 1 full-time faculty is at least 2 on an annual average 9 (2 out 40 medical schools fulfilled it). Item 92: Considering the number of people needed in Korea in areas such as basic medicine or health administration, 5% or more of annual admissions have advanced to such areas in10 to 20 years after graduation (No medical school fulfilled it).

### Correlation between scores by classical test theory and measure (difficulty parameter) by Rasch model of the items

**Fig. 1.**
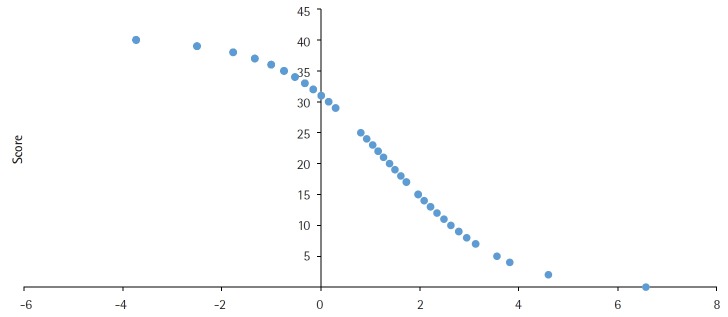
Correlation between the score obtained using classical test theory and measure (difficulty parameter) obtained using the Rasch model for the items (number of items=109; r=-0.9427; 95% confidence interval, -0.9605 to -0.0.9172; degrees of freedom=107; t=1.9824; P<0.0001).

A negative correlation was found (number of items=109; r=-0.9427; 95% confidence interval [CI], -0.9605 to -0.0.9172; degrees of freedom [df]=107; t=1.9824; P<0.0001) (Fig. 1).

### Correlation between scores and measure (difficulty parameter) of the 40 schools

**Fig. 2.**
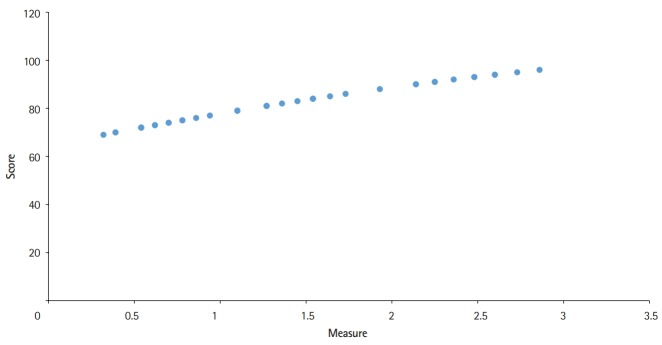
Correlation between the score obtained using classical test theory and measure (difficulty parameter) obtained using the Rasch model for 40 medical schools in Korea (number of items=40; 95% confidence interval, 0.09948 to 0.9986; r=0.9973; degrees of freedom=38; t=2.0244; P<0.0001).

A positive correlation was found (number of items=40; 95% CI, 0.09948 to -0.9986; r=0.9973; df=38; t=2.0244; P<0.0001) (Fig. 2).

### Goodness-of-fit test

Two of the 109 items had an outfit mean square exceeding 2.0 (Datasets 2, 5). The other 107 items were in the acceptable range of goodness of fit. All items had an infit mean square in the acceptable range (more than 0.5). Furthermore, 1 school had an outfit mean square exceeding 2.0. All schools were in the acceptable range for the infit mean square (more than 0.5) (Dataset 7).

## Discussion

### Key results

Out of 41 required items, average score was 30.0 (Dataset 6). The correlation between score and measure of 109 items was negative (r=-0.9427); meanwhile, correlation between score and measure of 40 medical schools was positive (r=0.9973). There were 2 outlier items and 1 outlier school. No items or schools were excessively well matched to the items or schools. Therefore, the 109 items used in the 2nd cycle of evaluations conducted from 2007 to 2011 for 40 medical schools by KIMEE can be said to fit the evaluation process.

### Interpretation and suggestions

Negative correlation between score and measure of items was already well-known phenomenon because the higher score showed lower measure. Positive correlation between score and measure of 40 medical school means that the latent trait of medical schools well presented as not only measure but also score.

“For the interpretation of the result of goodness of fit test, infit means the inlier-sensitive. It occurs not only when the examinees' responses are too suitable to the estimated response pattern but also their responses are least suitable to alternative evaluation tool. Outfit means outlier-sensitive. It occurred when the examinees' responses were far from expected ones. Outfit mean square value over 2.0 may distort measurement system (tool); while infit mean square value less than 0.5 may produce too higher reliability of the measurement system” [4].

The 2 outlier items were as follows:

The first outlier item (item 3) was “The college must have education, research and patient care policies regarding social accountability and such policies must be practiced”. Of the 40 schools, 39 fulfilled this item. Therefore, this item was too easy to fulfill, and the response was extraordinary, with a measure (difficulty parameter) of –2.5 (Dataset 7). The school that did not fulfil this item received a score of 88 out of 109.

The second outlier item (item 66) was “The ratio of faculty members who graduated from the same college is 70% or less among the total faculty of the medical school”. Its score was 31 out of 40, with a measure (difficulty parameter) of –0.01, meaning that this item had a central position of item difficulty. Therefore, many schools that could not fulfill this item received a higher or lower overall mark.

Least fulfilled items especially in required items listed in the results is suggested to be overcome by medical schools up to the next cycle of the evaluation and accreditation by KIMEE.

This study is the first analysis of the goodness of fit of items used for the evaluation and accreditation of medical schools in Korea. The results were favorable. Therefore, the evaluation and accreditation tools used for the 40 medical schools in the cycle from 2007 to 2011 were acceptable. It is necessary to check the psychometric properties of evaluation items regularly. Before the next cycle of evaluation and accreditation by KIMEE, non-fulfilled items should be stressed; furthermore, the outlier items should be re-considered to revise or exclude them.

### Limitations

Too many items (18 out of 109) were fulfilled by all schools. Therefore, the analysis by Winsteps was done without consideration of those items with a perfect score. Nonetheless, those items are necessary to assess whether medical schools are fulfilling their basic responsibilities; therefore, it would be impossible to remove or modify them for the purpose of improving the item analysis process. DIMTEST and DETECT were used for unidimensionality test, because they are no-parametric test in which case, the number of examinees (schools) is not a problem. Although the unidimensionality test was done with nonparametric methods, the number of examinees (schools) was too small (40) relative to the higher number of items (109). Therefore, it would be difficult to confirm that unidimensionality was appropriately tested. There has been no previous report of unidimensionality where the number of items exceeded the number of examinees. Although the assumption of unidimensionality was difficult to verify in this case, the items all focused on the evaluation and accreditation of medical schools. Therefore, the analysis was conducted according to the Rasch model; the results should be interpreted with this limitation in mind.

### Generalizability

This study presents data on all medical schools except one from a single country. It can represent the situation in Korea without difficulty. Since the content of items may vary from country to country, it is difficult to extrapolate the above results and interpretation to the accreditation of medical schools in other countries. Furthermore, it may be difficult to test unidimensionality due to the small number of examinees (medical schools) in a specific country. If the same accreditation items were to be introduced for medical schools throughout the world, it may become possible to test unidimensionality more confidently. Furthermore, the comparison among medical schools in different countries may be possible.

### Conclusion

This goodness-of-fit analysis based on the Rasch model showed that the evaluation items used in the 2nd cycle of evaluation and accreditation of 40 medical schools from 2007 to 2011 by KIMEE were favorable. One of the 40 schools was an outlier in terms of its responses to items; therefore, it is necessary to determine what the problem was regarding the responses at this school.

## Footnotes

**Data availability** Data files are available from Harvard Dataverse: https://doi.org/10.7910/DVN/T6WC1T Dataset 1. Dichotomous data converted from raw data of the items used in the 2nd cycle of evaluation and accreditation of medical schools by the Korea Institute of Medical Education and Evaluation from 2007 to 2011. Dataset 2. Content of the items used in the 2nd cycle evaluation and accreditation. Dataset 3. Results of Winsteps. Dataset 4. Results of DIMTEST. Dataset 5. Results of DETECT. Dataset 6. Results of goodness-of-fit testing for items. Dataset 7. Results of goodness-of-fit testing for schools.

## Supplementary materials

Supplementary materials are available from Harvard Dataverse: https://doi.org/10.7910/DVN/T6WC1T

Supplement 1. Audio recording of the abstract.
